# Up to 7% of referrals to oral and maxillofacial surgery are related to medication-related osteonecrosis of the jaws: how much is really out there?

**DOI:** 10.1038/s41405-022-00097-6

**Published:** 2022-02-22

**Authors:** Ailish Clark, Iain Pretty

**Affiliations:** 1 Department of Oral Surgery, University Dental Hospital Manchester, Manchester Foundation Trust & Division of Dentistry, University of Manchester, Manchester, UK; 2 Division of Dentistry, University of Manchester, Manchester, UK

**Keywords:** Oral diseases, Osteonecrosis of the jaw

## Abstract

**Introduction:**

Medication-related osteonecrosis of the jaws (MRONJ) is generally described as rare; therefore, firm incidence data are challenging to ascertain.

**Aim:**

Using two sites in Northwest England, ascertain the number of referrals to oral and maxillofacial surgery involving:

- Suspected MRONJ.
- Patients at risk of MRONJ requiring a dentoalveolar procedure.

**Method:**

All sequential referrals over a 2-year period were analysed. The referrals were categorised into ‘type’ of referral (stage 1). Any referral for MRONJ, or patient at risk, was then further examined (stage 2).

**Results:**

A total of 2150 referrals were screened. The most common referral reasons were temporomandibular joint issues and hard tissue conditions. The proportion of referrals for suspected MRONJ was similar for both sites: 3.7% (site 1) and 3.4% (site 2). At site 1, 1.6% of all referrals were at risk of MRONJ referred for treatment. In site 2, 3.8% of all referrals were in this category.

**Conclusion:**

Despite limitations, the finding that patients with or at risk of MRONJ potentially equates to 7% of all referrals represents a substantial proportion of OMFS practice. Therefore, there are clear benefits of collecting accurate data prospectively to understand the scale of this condition and its impact on services.

## Introduction

Medication-related osteonecrosis of the jaws (MRONJ) was first described in 2003 by Marx et al. [1]. Since 2003, awareness of MRONJ has grown exponentially with countless publications on the subject. The nomenclature has also evolved from bisphosphonate-related ONJ to antiresorptive-related ONJ to MRONJ, reflecting the implication of drugs other than bisphosphonates in the development of MRONJ, namely antiresorptive and some antiangiogenic medications [2].

MRONJ is generally described as rare; however, it is not an uncommon presentation to oral surgery and oral and maxillofacial surgery units [2–7]. Despite this, firm incidence (and hence prevalence) data are hard to ascertain. The risk of MRONJ is largely estimated at less than 1% of those at risk [8] and influenced by numerous factors such as indication for the implicated drug, modality of drug, extent of any dentoalveolar surgery carried out and co-morbidities, etc. [9, 10].

Attempts to ascertain national UK MRONJ incidence data have proved challenging due to difficulties collecting source data from surgical units [9]. In addition, cases may be misdiagnosed as other pathologies such as osteomyelitis and therefore omitted from data collection. Fedele et al. also discussed that patients with the non-exposed bone variant of MRONJ may be missed in MRONJ case data collection, again leading to incorrect incidence data [10]. There are some large clinical trials of specific drugs reporting incidence of MRONJ; however, strict exclusion of patients requiring dental treatment mean results may not be generalisable [11].

As numbers are low, cases are often seen sporadically by a range of clinicians across a large number of units. Without the mechanism to collate these data, the true number of MRONJ cases is currently unknown. Furthermore, due to the lack of agreed consensus on managing MRONJ patients, case management is variable, as is disease resolution. Treatments can range from simple symptom management up to large resection and subsequent reconstruction. These factors may potentially have a huge impact on service planning, provision of care and resource management. Therefore, there is a clear need to establish the firm incidence and outcome data relating to different management strategies in this country, a comment echoed by Cochrane, SDCEP and AAOMS [2, 8, 12].

## Aim

To estimate the number of possible MRONJ cases being referred to oral surgery/oral and maxillofacial surgery using two sites in Northwest England, and identify and analyse all referrals involving:

- Suspected MRONJ cases.
- Patients at risk of developing MRONJ requiring a dentoalveolar procedure.

## Method

Referral data at the source of initial receipt were examined to explore the extent of potential MRONJ cases and those at risk of MRONJ. Two sites were used for analysis: site 1 is a teaching hospital (TH) and site 2 is a district general hospital (DGH). All sequential referrals to both sites from 1 November 2016 for a period of 2 years were collected.

An automated search was considered to screen large numbers of referrals but dismissed as many referrals were handwritten. Therefore, referrals were hand searched and screened in totality to avoid omissions. Referrals were analysed in two stages described below. Referrals were fully anonymised prior to assessment in compliance with the ICO guidelines on secondary use data and the study was undertaken under a service evaluation model hence ethical approval was not required.

### Stage 1—Referral overview and screening

Referrals were categorised into ‘type’ of referral under broad headings including hard tissue (such as dentoalveolar procedures), soft tissue, oral medicine (oro-mucosal conditions, facial pain), salivary gland, temporomandibular joint complaints and miscellaneous (for any others). This provided an overview of the service in each unit based on the distribution of types of referrals received. Each referral was reviewed in entirety regardless of referral type to avoid omission of possible cases.

Any potential referral for MRONJ or patient at risk of MRONJ requiring surgical intervention was selected for further analysis in stage 2.

### Stage 2—Referral refinement for possible MRONJ cases/patients at risk of MRONJ

Potential MRONJ referrals (either with a possible diagnosis or those at risk of developing MRONJ who required a surgical procedure) from any referral type were then further examined and the following information extracted—sex, age in years, nature of referral and description included in the referral for possible MRONJ cases.

## Results

### Stage 1—Referral overview and screening

A total of 2150 referrals were screened, 1500 from site 1 (TH) and 650 from site 2 (DGH). The discrepancy in numbers reflects the size of the units and geographical footprint served, hence the difference in volumes of referrals during this period.

After removal of duplicates, 1465 referrals to the TH and 649 referrals to the DGH remained. Stage 1 analysis can be seen in Table 1.

**Table 1:** Nature of referrals to teaching hospital and district general hospital.

Nature of referral	Teaching hospital (TH)		District general hospital (DGH)	
	Number	% frequency	Number	% frequency
Hard tissue	**557**	**38**	**298**	**46**
Soft tissue	115	8	117	18
TMD	**664**	**45**	**175**	**27**
Oral medicine	89	6	33	5
Salivary gland	24	2	18	3
Miscellaneous	15	1	4	1
TMD and hard tissue	0	0	4	1
Total	1465		649	

The numbers in bold are referral type in highest proportion from each site.

Within the TH site, the most common referral reason was temporomandibular joint issues, 45% of all referrals (66 patients). In the DGH site, the most common reason for referral was hard tissue conditions, 46% of all referrals (298 patients).

### Stage 2—Referral refinement for possible MRONJ cases/patients at risk of MRONJ

All possible cases of MRONJ or patients at risk of developing MRONJ were further analysed as two separate categories and the following data were extracted: referral type, clinical description, age and sex.

**Fig. 1:**
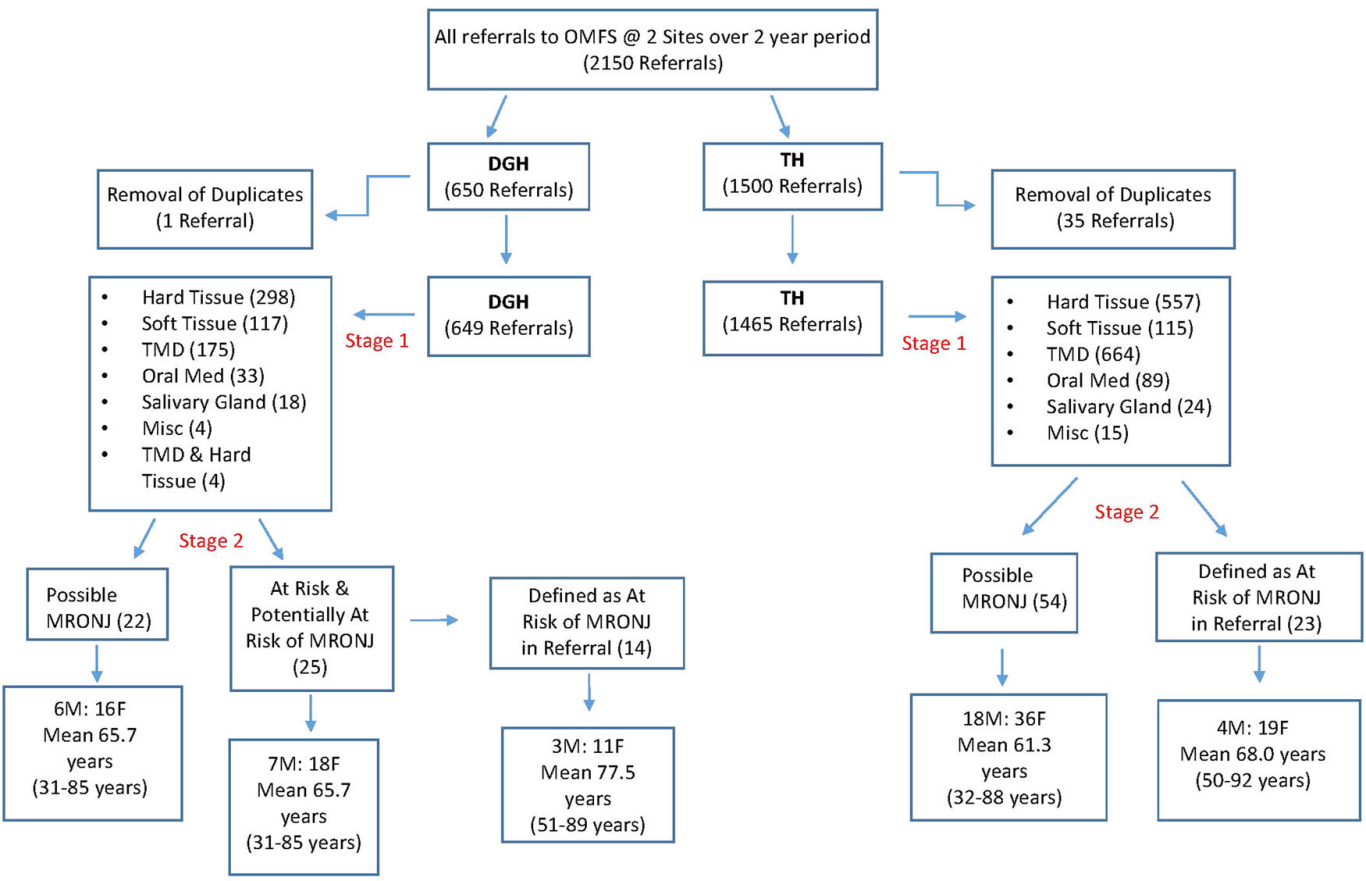
Flowchart of search and analysis of OMFS referrals. The following chart illustrates the analysis of referrals from both sites at stage 1 and stage 2 highlighting the main findings from each stage. M Male, F Female, (number of referrals or age - defined within box).

A diagrammatic illustration of the search can be seen in Fig. 1.

### Referrals of possible MRONJ cases

There were 54 referrals for possible/established MRONJ to the TH accounting for 3.7% of all referrals. There were 22 referrals for possible MRONJ to the DGH, accounting for 3.4% of all referrals. The features noted in the referrals are illustrated in Table 2. In TH referrals for possible MRONJ, the most common feature described was osteonecrosis/MRONJ (37%, 20 cases) or poorly healing socket (30%, 16 cases). The most common reported feature in DGH referrals was exposed bone (23%, 5 cases) or pain after extraction (18%, 4 cases). Interestingly, those referred for suspected MRONJ made up 3% of all referrals to both sites, representing a significant proportion of new patients.

**Table 2:** Features reported in referrals of possible MRONJ cases.

Feature reported in referral	Teaching hospital (TH)		District general hospital (DGH)	
	Number	% frequency	Number	% frequency
Poorly healing socket	**16**	**30**	3	13
Possible osteonecrosis/MRONJ	**20**	**37**	3	13
Pain after extraction	5	9	4	18
Exposed bone	5	9	**5**	**23**
Swelling and edentulous	3	4	1	5
Pain and edentulous	1	2	1	5
Vague^a^	2	4	**5**	**23**
Unusual radiolucency	2	4	0	0
Total	54		22	

^a^Vague: swelling, suppuration altered sensation +/− including history of bisphosphonates/osteoporosis.

The numbers in bold are referral type in highest proportion from each site.

Most patients referred for possible MRONJ to the DGH were female, 73% (16 patients), mean age was 65.7 years (range 31–85 years). Those referred to the TH had a similar gender distribution: female (66%, 36 patients) vs male (32%,18 patients). The average age was slightly lower at 61.3 years (range 32–88 years).

### Referrals for dentoalveolar surgery in patients at risk of MRONJ

A total of 1.6% (23 patients) of all TH referrals were patients at risk of MRONJ referred for treatment (i.e., extractions or dentoalveolar surgery). The majority were female, 82% (19 patients), mean age was 68 years (range 50–92 years).

A total of 3.8% (25 patients) of all DGH referrals were in this category comprising of 11 patients noted as ‘at risk’ in the referral, and an additional 14 patients possibly at risk (from features in referral). In all, 72% (18 patients) were female and 28% were male (7 patients), mean age was 75 years (age range 51–89 years). It was noted that those referred to OMFS DGH had an older profile in both the ‘at risk’ and the ‘suspected’ MRONJ groups than those referred to the TH.

When analysing the DGH referrals, there were several patients identified as ‘possibly at risk’ by the reviewer based on features documented in the referral. These patients were not explicitly stated as at ‘risk of MRONJ’ in the referral; however, the information provided from the referrer indicated a possible risk of MRONJ. This included, for example, a patient with osteoporosis but with no medication history included in the referral. Therefore, these referrals were classified as ‘possibly at risk’ in the results section. For the TH patients, none of the referrals were identified in this grouping. The only ‘at risk’ referrals included for the TH implicitly stated that the patient was at risk of MRONJ.

## Discussion

### Overview

The most common ‘type’ of OMFS referral to both units was hard tissue conditions and temporomandibular joint issues. The distribution however varied between the sites, likely due to the organisational setup of these services. Within the surrounding area of the DGH there may be fewer tier 2 services offering dentoalveolar surgery, hence, the large number of referrals for this type of treatment to OMFS. Tier 2 oral surgery services are a commissioned service in England that carry out oral surgery procedures on referral from primary care. These cases are deemed too complex for general dental practice and therefore require a practitioner with additional skills. The referral practices that commonly refer to the TH have access to a large number of dentoalveolar surgical services (tier 2) and therefore less referrals of this type are referred into the hospital. In addition, this unit offers a dedicated TMD clinic, an example of possible provider-induced demand seen in the referral profile.

### MRONJ

Interestingly, the proportion of referrals for suspected MRONJ was similar for both sites; 3.7% of all referrals to TH and 3.4% to DGH. Considering the volume of new OMFS referrals each month, this equates to a substantial proportion of the workload. In addition, potentially 1–2% of all referrals to OMFS were for the treatment of patients at risk of developing MRONJ. This figure is similar to salivary gland pathology referrals, 2.7% DGH vs 1.6% TH, which is considered very much part of normal OMFS practice.

One study based at a DGH OMFS site reported that from a sample of 58 patients at risk of MRONJ who had dentoalveolar surgery, 20 subsequently developed MRONJ (approximately 35%) [13]. These patients were a mixture of high and low risk patients taking antiresorptive medications for various cancers and osteoporosis in IV and oral form, the majority in the latter group (13 patients) [13]. With 3 million people in the UK with osteoporosis and nearly 5000 new cases of multiple myeloma diagnosed each year, just two groups potentially at risk of MRONJ due to their medical management, the pool of patients at risk of MRONJ is potentially huge [14, 15]. If 35% of those referred patients at risk go on to develop MRONJ, the case load and burden of care could be substantial. One study in the US found that the median cost of managing a case of MRONJ was $1667, but could extend into $10,000s, which adds up to a costly portion of the services managing these patients [16].

Whilst the above may be alarming, it is well reported that capturing data on rare conditions, such as MRONJ, is complex, due to variations in reporting, descriptors of symptoms and diagnostic criteria, etc. [17]. Therefore, we do not know if the above is a true representation of the level of MRONJ throughout the country. There have been attempts to collect data elsewhere around the world using databases of other conditions as surrogate markers, for example, osteomyelitis or inflammatory conditions of the bone, and extrapolating data, but this poses the risk of incorrectly identifying cases. Ideally, it would be possible to search diagnostic coding databases for confirmed cases of MRONJ using for example SNOMED, a clinical coding system utilised in the NHS, to gain access to a much larger meaningful data sample and draw meaningful conclusions. At present, however, an individual diagnostic code for this condition does not exist and currently, there is no national data on this condition. So, the question remains, just how much MRONJ are we seeing in the UK and what is the impact on oral surgery and oral and maxillofacial services both logistically and financially?

### Limitations

This review of referrals has several limitations, the referrals were hand screened and analysis involved some discretion to include all possible cases of MRONJ. For example, a referral for a poorly healing socket in an elderly patient with osteoporosis but no note of MRONJ/antiresorptive use may very well be a case of MRONJ when further explored and therefore was included as a possible case. Also due to the inclusion of handwritten referrals, an automated screening process was not possible which could have increased the number of referrals included in a shorter time frame with greater efficiency. Even with a pool of 2000+ potential patients, with an estimated incidence of MRONJ of less than 1%, the assessed sample is small. Furthermore, the hand searching was carried out by one assessor that could lead to over or under inclusion of cases.

As the information in this analysis was extracted solely from that provided in referral documents from primary care clinicians, there are other potential confounders. It is possible that potential patients were omitted incorrectly due to lack of detail in referral letters or that patients were included incorrectly due to the same reason, again causing the analysis to be over- or underinclusive. One study of referral letters to OMFS services found that 70% of all referrals were missing at least one clinically relevant detail, highlighting that there could be far more patients suitable for inclusion that have been missed [18].

Due to the anonymised nature of this service evaluation, it was not possible to follow the patient journey and determine the final diagnosis and outcome of those referred for possible MRONJ or those at-risk requiring treatment. Therefore, we do not have true incidence data for this sample.

### Further research

Despite the limitations of this study, the finding that patients at risk of MRONJ and those with suspected MRONJ potentially equates to up to 7% of all referrals to OS/OMFS services, highlights that MRONJ may represent a much larger part of practice than previously considered. Whilst it cannot be established from this work how many of these cases went on to develop MRONJ, the volume of new referrals alone for this condition clearly form a significant proportion of new referrals to OMFS and OS. Every new referral requires at least a consultation appointment, plus potential treatment. For example, a patient at risk of MRONJ will likely require a consultation, an appointment for dentoalveolar surgery followed by at least one review to ensure adequate healing. This highlights a potentially huge burden of care on these surgical services.

It is well recognised that there are several unanswered questions surrounding MRONJ due to its apparent rarity and the corresponding difficulty with collection on a large scale highlighted earlier. The need for collation of national data on MRONJ was also raised in a paper by Gliklich in 2009 and benefits include; mapping out the natural history of the disease, analysis of interventions and outcomes and ultimately improving quality of life [19]. Therefore, there are clear benefits to patients and clinicians in collecting accurate prospective data to understand the true incidence of MRONJ and the impact on our services.

As a result, a prospective multisite registry is currently in development to be rolled out for use across the UK. MRONJ lends itself well to a database due to the established classification and staging criteria [2]. In addition, due to the nature of the condition, patients are highly likely to be sent to a limited number of specialties; mainly oral surgery and oral and maxillofacial surgery. This consolidates the patient journey and hopefully lends itself to the collection of this data.

## Conclusion

By collating much larger data sets on MRONJ cases than has previously been possible in the UK, patterns may emerge, informing decision making regarding these patients and ultimately improve patient care. Only then, when we have begun to combine this data on a wide scale will we be able to assess the true extent of MRONJ and design services accordingly to tackle this difficult to treat condition.

## References

[1] Marx RE (2003). Pamidronate (Aredia) and zoledronate (Zometa) induced avascular necrosis of the jaws: a growing epidemic. J Oral Maxillofac Surg..

[2] Ruggiero SL, Dodson TB, Fantasia J, Goodday R, Aghaloo T, Mehrotra B (2014). American Association of Oral and Maxillofacial Surgeons position paper on medication-related osteonecrosis of the jaw-2014 update. J Oral Maxillofac Surg..

[3] de Boissieu P, Gaboriau L, Morel A, Trenque T (2016). Bisphosphonate-related osteonecrosis of the jaw: data from the French national pharmacovigilance database. Fundam Clin Pharmacol..

[4] Eiken PA, Prieto-Alhambra D, Eastell R, Abrahamsen B (2017). Surgically treated osteonecrosis and osteomyelitis of the jaw and oral cavity in patients highly adherent to alendronate treatment: a nationwide user-only cohort study including over 60,000 alendronate users. Osteoporos Int..

[5] Iwamoto J, Uzawa M (2016). Experience with alendronate treatment for 7 years among Japanese men with osteoporosis or osteopenia and clinical risk factors for fractures. Clin Rheumatol..

[6] Kwon JW, Park EJ, Jung SY, Sohn HS, Ryu H, Suh HS (2015). A large national cohort study of the association between bisphosphonates and osteonecrosis of the jaw in patients with osteoporosis: a nested case-control study. J Dent Res..

[7] Sammut S, Malden N, Lopes V, Ralston S (2016). Epidemiological study of alendronate-related osteonecrosis of the jaw in the southeast of Scotland. Br J Oral Maxillofac Surg..

[8] Scottish Dental Clinical Effectiveness Programme. SDCEP oral health management of patient at risk of MRONJ guidance: SDCEP. Updated 2017. http://www.sdcep.org.uk/wp-content/uploads/2017/04/SDCEP-Oral-Health-Management-of-Patients-at-Risk-of-MRONJ-Guidance-full.pdf. Accessed 15 Jan 2019.

[9] Rogers SN, Palmer NOA, Lowe D, Randall C (2015). United Kingdom nationwide study of avascular necrosis of the jaws including bisphosphonate-related necrosis. Br J Oral Maxillofac Surg..

[10] Fedele S, Bedogni G, Scoletta M, Favia G, Colella G, Agrillo A (2015). Up to a quarter of patients with osteonecrosis of the jaw associated with antiresorptive agents remain undiagnosed. Br J Oral Maxillofac Surg..

[11] Boquete-Castro A, Gomez-Moreno G, Calvo-Guirado JL, Aguilar-Salvatierra A, Delgado-Ruiz RA (2016). Denosumab and osteonecrosis of the jaw. A systematic analysis of events reported in clinical trials. Clin Oral Implants Res..

[12] Beth-Tasdogan NH, Mayer B, Hussein H, Zolk O (2017). Interventions for managing medication-related osteonecrosis of the jaw. Cochrane Database Syst Rev..

[13] Bhanji AH. Medication related osteonecrosis of the jaws: review of care at Royal Albert and Edward Infirmary. BAOMS; 2017.

[14] NHS. Osteoporosis. 2020. https://www.nhs.uk/conditions/osteoporosis/. Accessed 1 Dec 2020.

[15] Inform N. Mutliple myeloma NHS inform website: NHS. 2020. NHS inform illness and conditions. https://www.nhsinform.scot/illnesses-and-conditions/cancer/cancer-types-in-adults/multiple-myeloma. Accessed 1 Dec 2020.

[16] Najm MS, Solomon DH, Woo SB, Treister NS (2014). Resource utilization in cancer patients with bisphosphonate-associated osteonecrosis of the jaw. Oral Dis..

[17] Auvin S, Irwin J, Abi-Aad P, Battersby A (2018). The problem of rarity: estimation of prevalence in rare disease. Value Health.

[18] DeAngelis AF, Chambers IG, Hall GM (2010). The accuracy of medical history information in referral letters. Aust Dent J..

[19] Gliklich R, Wilson J (2009). Epidemiology of bisphosphonate-related osteonecrosis of the jaws: the utility of a national registry. J Oral Maxillofac Surg..

